# Determination of Microalgal Lipid Content and Fatty Acid for Biofuel Production

**DOI:** 10.1155/2018/1503126

**Published:** 2018-05-21

**Authors:** Zhipeng Chen, Lingfeng Wang, Shuang Qiu, Shijian Ge

**Affiliations:** Jiangsu Key Laboratory of Chemical Pollution Control and Resources Reuse, School of Environmental and Biological Engineering, Nanjing University of Science and Technology, Xiao Ling Wei 200, Nanjing, Jiangsu 210094, China

## Abstract

Biofuels produced from microalgal biomass have received growing worldwide recognition as promising alternatives to conventional petroleum-derived fuels. Among the processes involved, the downstream refinement process for the extraction of lipids from biomass greatly influences the sustainability and efficiency of the entire biofuel system. This review summarizes and compares the current techniques for the extraction and measurement of microalgal lipids, including the gravimetric methods using organic solvents, CO_2_-based solvents, ionic liquids and switchable solvents, Nile red lipid visualization method, sulfo-phospho-vanillin method, and the thin-layer chromatography method. Each method has its own competitive advantages and disadvantages. For example, the organic solvents-based gravimetric method is mostly used and frequently employed as a reference standard to validate other methods, but it requires large amounts of samples and is time-consuming and expensive to recover solvents also with low selectivity towards desired products. The pretreatment approaches which aimed to disrupt cells and support subsequent lipid extraction through bead beating, microwave, ultrasonication, chemical methods, and enzymatic disruption are also introduced. Moreover, the principles and procedures for the production and quantification of fatty acids are finally described in detail, involving the preparation of fatty acid methyl esters and their quantification and composition analysis by gas chromatography.

## 1. Introduction

Nowadays, limited stock of petroleum-derived fuel resources combined with perpetually increasing demands for energy due to the rapid industrialization and population growth has troubled many governments and organizations across the world [1]. Moreover, the combustion of fossil-derived fuels has led to increasing emission of greenhouse gases such as carbon dioxide (CO_2_), leading to global climate change and posing threats to the biosphere [2]. In order to achieve sustainable development, the critical issues noted above and the gradually rising fossil-derived fuel prices have called for the needs to search for alternative sustainable and renewable energy sources [3].

Biofuels, produced from biomass, are promising alternatives to fossil-derived fuels due to several distinct advantages including carbon neutrality, reduced emissions of gaseous pollutants (e.g., carbon monoxide, CO_2_, and sulfur oxides), continuous availability of biomass feedstocks, and their safety of production by farming [4]. According to their physical characteristics, biofuels are divided into solid (i.e., biochar), liquid (i.e., bioethanol, vegetable oil, and biodiesel), and gaseous (i.e., biogas, biosyngas, and biohydrogen) fuels. Based on the types of used feedstocks, biofuels are categorized into three generations. The first generation feedstocks mainly include food crops such as corn, soybean, rapeseed, sunflower, and palm oil. The second-generation biofuels are derived from nonedible feedstocks like* Jatropha*,* Miscanthus*, Switch grass, and other organic wastes. Nevertheless, the expanding demand for edible feedstocks as food sources and their need for large areas of arable land for production have limited the development of both the first- and second-generation biofuels. The use of microalgae as a third-generation biofuel feedstock avoids these issues and presents several distinct advantages of not requiring agricultural or arable lands for production, high photosynthetic efficiencies and biomass productivities (biomass doubled in less than one day), and 100 times more lipids per acre of land [5, 6]. Moreover, the main storage lipids in microalgae are neutral lipids (NLs) or triacylglycerols that can be esterified to FAMEs with the primary profiles of C16 and C18, proven to be the most suitable for biofuel production [7]. Microalgae exhibit great adaption to various environmental conditions, making them easy to cultivate. For instance, they can grow on marginal land in both the open pond and closed systems using waste streams like wastewater, waste or CO_2_-enriched gas (biogas, flue gas), waste organics (i.e., crude glycerol), and waste heat to provide nutrients and carbon and temperature maintenance (in a cold climate), achieving economically feasible and environmentally sustainable biofuel production and waste bioremediation [8]. Moreover, various routes of microalgal metabolisms can be adopted for enhanced growth and lipid production. Traditionally, phototrophic algae are grown autotrophically with CO_2_ as the unique carbon source and light providing all the energy needed. Moreover, some microalgae species can grow heterotrophically using only organic compounds, while others can grow mixotrophically using both organic compounds and CO_2_ to support growth [9].

Currently, the high costs of the important microalgal harvesting and lipid extraction processes are the primary obstacles impeding on the commercial application of microalgae-derived biofuel production [10–12]. For example, lipid extraction is a high-power-consumption process because lipids are stored in microalgal cells and the cell wall is a thick and rigid layer composed of complex carbohydrates and glycoproteins with high mechanical strength and chemical resistance, posing difficulties for lipid extraction [13]. Therefore, certain cell disruption techniques are generally considered prior to lipid extraction to improve the extraction efficiencies. Nevertheless, the efficiencies of cell disruption and lipid extraction vary with methods selected with different operating conditions (e.g., temperature, atmospheric pressure, and humidity), microalgae species, and biomass amount.

This review summarizes and compares the methodologies employed for the extraction and quantification of microalgal lipids. The pretreatment methods supporting cell disruption and subsequent lipid extraction are also included. Finally, the principles and procedures for the production and quantification of fatty acids in microalgae are discussed in detail.

## 2. Microalgal Lipids

**Figure 1:**
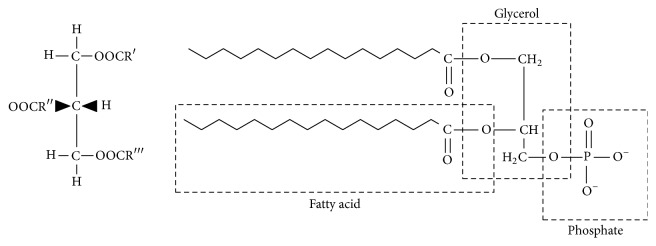
Lipid molecules. Triacylglycerol (NL) on the left. Phospholipid (polar lipid) on the right. R′, R′′, and R*‴* in the triacylglycerol molecule represent fatty acid chains. Phospholipid molecule is negatively charged [15].

Microalgal lipids can be divided into two groups according to their structures: nonpolar NLs (acylglycerols, sterols, free fatty acids, wax, and steryl esters) and polar lipids (phosphoglycerides, glycosylglycerides, and sphingolipids).  Figure 1 shows the structural formula of the polar lipid and NLs [14, 15]. These lipids play different but important roles in microalgal metabolism and growth period. Some lipids such as phosphoglycerides, glycosylglycerides, and sterols are imperative structural components of biological membranes, while lipids like inositol lipids, sphingolipids, and oxidative products of polyunsaturated fatty acids may act as key intermediates in the cell signaling pathways and play a role in sensing changes in the environment [16]. The quantities of these microalgal lipids vary with the type of species, growth conditions, and ambient environments. It was reported that the lipid contents ranged at 20–50% of dry biomass including* Chlorella*,* Crypthecodinium*,* Cylindrotheca*,* Dunaliella*,* Isochrysis*,* Nannochloris*,* Nannochloropsis*,* Neochloris*,* Nitzschia*,* Phaeodactylum*,* Porphyridium*,* Schizochytrium*, and* Tetraselmis *[17].

## 3. Microalgal Cell Disruption Methods

Specific microalgal cell pretreatment procedures must be considered prior to the subsequent lipid extraction due to the microalgal cell wall structure. When the extraction is conducted from the wet biomass, the pretreatment step is mandatory to disrupt the microalgal cell walls and allow the lipids to be released into the extracting mixture. The commonly used pretreatment methods are summarized below.

### 3.1. Bead Beating

Bead beating, also known as bead mill or ball mill, disrupts cells by the impact of high-speed spinning of fine beads on the biomass slurry. The whole disruption process could be done within minutes, and it could be applied to any kinds of microalgae without preparation [18, 19]. Two common types of bead mills are shaking vessels and agitated beads [20]. Shaking vessels usually consist of multiple containers or well-plates on a vibrating platform, and the cell disruption is done by shaking the entire vessel on a vibrating platform. The shaking vessels are usually employed in laboratory, as they are only suitable for multiple samples requiring similar disruption treatment conditions. Comparatively, the agitated beads type that is made up of a rotating agitator in a fixed vessel filled with beads and cell culture could achieve better disruption efficiencies. However, the cooling jackets must be equipped to protect the heat-sensitive biomolecules as the rotating agitator generates heat during disruption process [18, 19]. The combination of agitation, collision, and grinding of the beads could produce a higher disruption efficiency [20]. To sum up, the simplicity of the equipment and the rapidness of the treatment process are the two main advantages of bead beating methods, while the requirement of an extensive cooling system to protect the target products has limited it to scale up [18].

### 3.2. Microwave

Microwave is an electromagnetic wave with the frequency ranging between 300 MHz and 300 GHz, which is lower than that of infrared and higher than that of radio waves. Microwave-assisted extraction technology has been studied for extracting target compounds in a few fields, including microalgal lipid extraction [50]. When microalgal cells are exposed to the microwave with the specific frequency (approximately 2450 MHz), cell molecules generate a rapid oscillation within the rapidly oscillating electric field, resulting in the heat generation due to the frictional forces from the inter- and intramolecular movements [51]. The intracellular heating causes the water to vapor, which disrupts the cells and subsequently opens up the cell membrane. This method exhibits strong advantages of short reaction time, low-operating costs, and efficient extraction with all of the species, but the requirement of a vast cooling system to protect target products limits its large-scale application [18, 19].

### 3.3. Ultrasonication

Ultrasonication has been a well-known method for the microbial cell disruption due to its short reaction time with high productivity [52]. When the ultrasound is applied to the liquid cultures, small “vacant regions” called microbubbles are momentarily formed as the liquid molecules are moved by the acoustic waves. Meanwhile, the production of microbubbles causes cavitation, which in turn creates pressure on the cells to break up [53]. During the treatment, the rapid compression/decompression cycles of the sonic waves generate transient and stable cavitation. The transient cavitation occurs when oscillations that cavitation undergoes are unsteady and implode ultimately. This type of implosion could produce extremely localized shock waves and high temperature, the conditions of which impart mechanical stress on the cells and crack the cell wall and membrane [54]. On the other hand, the cavitation that oscillates for many cycles is referred to as stable cavitation, which can produce microscale eddies, inducing stress or physiological changes in microorganisms [55]. Ultrasonic horn and bath are the two basic types of sonicators, and they are commonly employed in batch operations but can also be adapted for continuous operations [56]. Horns use piezoelectric generators, which are made of lead zirconate titanate crystals and vibrate with amplitude of 10–15 mm. As the energy generated at the horn tip dissipates rapidly with distance, the cavitation must be created with sufficient disruptive force. Transducers placed at the bottom of the sonicator are used in sonicator baths to generate ultrasonic waves. In sonicator baths, the number and arrangement of transducers vary according to the capacity and shape of the sonicator [20]. The working conditions of ultrasonication treatment are easy to set up and the whole process could be done in a very short time while with high reproducibility [19]. However, it is difficult to scale up as cavitation, the strong effect of which is able to achieve cell disruption only occurs in small regions near ultrasonic probes.

### 3.4. Chemical Method

The rupture of cells occurs when chemicals are used to increase the permeability of cell up to a particular value [57]. It was reported that, through chemical treatments with acids (i.e., HCl and H_2_SO_4_), alkalis (i.e., NaOH), and surfactants, chemical linkages on the microalgal cell envelope were degraded followed by the lysis of cell wall [58]. Comparatively, chemical treatment consumes less energy because it does not require a large amount of heat or electricity while showing higher efficiency of cell disruption. However, the continuous consumption of chemicals challenges the economic sustainability of this method. Moreover, acids and alkalis have a high risk of corroding the reactor and attacking microalgal lipids, thus ruining the whole process [18].

### 3.5. Enzymatic Disruption

In addition to the autolysis, the use of foreign lytic enzymes is extensively investigated because enzymes are the commercially available and easily controlled biological materials [59]. Specific enzyme is able to degrade certain structural cell components, thus improving the release of desired intracellular compounds [60]. In some cases, a mixture of different enzymes is reported to have a better economic and technical feasibility, and the lipid yields could be improved when enzymatic hydrolysis is combined with acid/alkaline pretreatment. Compared to the chemical method that possibly destroys every particle existing in the solution and even induces side-reactions of the target products (i.e., lipids), the reaction condition of enzymatic method is mild, and its selectivity is high with specific chemical linkages. Moreover, enzymatic disruption combined with other methods is usually considered for economic process and improved disruption performance [18]. However, more researches need to be conducted to reduce the high cost and relatively long treatment time, which have limited the large-scale application of this method.

### 3.6. Other Methods

**Figure 2:**
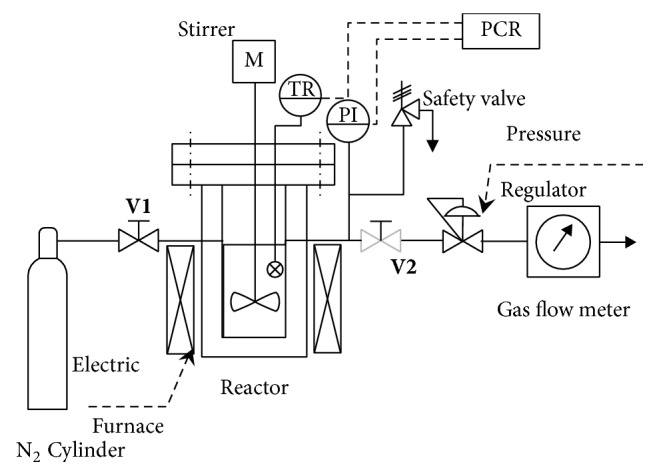
Schematic diagram of thermal pretreatment apparatus [61].

Apart from the methods noted above, there are some other microalgal cell disruption approaches that have been investigated as well. For example, when the mixture of microalgae and other solvents is sprayed through a narrow tube under high pressure, hydraulic shear force is generated and high pressure homogenization (HPH), also known as French press, makes use of this force to extract internal substances of microalgae. By measuring the increase in the soluble chemical oxygen demand (SCOD) during the cell disruption, lots of researchers have evaluated the cell disruption efficiency and found that HPH exhibited high cell disruption efficiency. It is worth mentioning that HPH has lots of advantages such as producing less heat during the extraction process, thus requiring less cooling cost and it is easy to scale up. However, the pretreatment process relying on HPH requires a relatively long time and consumes quite a few amount of power. Electroporation can achieve permanent cell disruption by applying a much stronger electromagnetic field (EF) to the biomass that can damage the cell envelopes beyond their healing abilities since the application of an EF of suitable intensity will lead to the formation of pores on the cell envelopes of the cells, and the pores are closed by a healing process when the EF is removed. Electroporation is a promising cell disruption method as it requires simple equipment and operation procedures with high energy efficiency [18, 20]. Thermal treatment could achieve effective recovery of hydrocarbons as well and a typical process of thermal pretreatment is shown in Figure 2. Firstly, place the samples in vessel and replace the air in the vessel by nitrogen gas. Then, heat vessel to the set temperature, and afterwards, cool the vessel to the ambient temperature after maintaining the samples at the set temperature. Then, stir the samples mechanically. Finally, open the autoclave and carefully remove the samples for further analysis [61]. Recently, plenty of work have been done to compare the efficiency of different cell disruption methods; however, due to the different species of microalgae used in experiments so as the different operating temperatures, atmospheric pressures, and other influence factors, the efficiency of different cell disruption methods is short of comparability.

## 4. Microalgal Lipid Extraction and Quantification Approach

The microalgal lipid extraction refers to the process of separating the valuable NLs and fatty acids from the cellular matrix and water. As far, multiple methods have been reported for the quantification of microalgal lipids, mainly including the conventional gravimetric method using extraction solvents, Nile red lipid visualization method, SPV, and TLC [62–64].

### 4.1. Gravimetric Method

The gravimetric method is most widely used to determine microalgal lipid content. It is also frequently used as a reference standard to validate other methods. The gravimetric method consists of the lipid extraction using solvents and lipid quantification achieved by recording the weight of extracted lipids after evaporating the extracting solvents. The extraction solvents used include the conventional organic solvents, CO_2_-based solvents, ionic liquids (ILs), and switchable solvent.

#### 4.1.1. Organic Solvent Extraction

**Figure 3:**
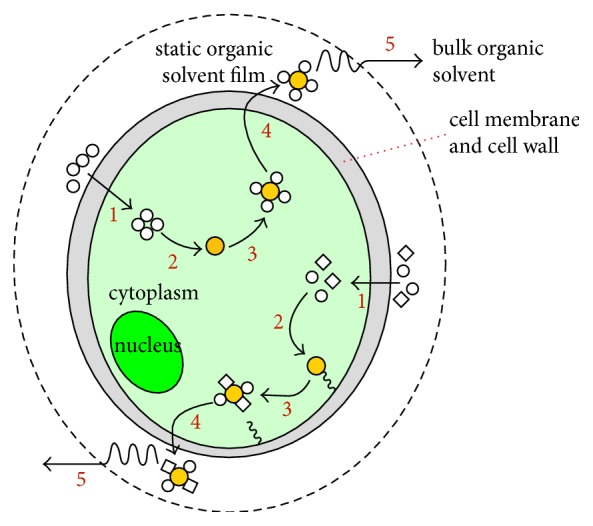
Schematic diagram of the organic solvent-based microalgal lipid extraction mechanisms. The pathway shown at the top of the cell is for nonpolar organic solvent while the pathway shown at the bottom of the cell is for nonpolar/polar organic solvent mixture. Orange circle: lipids, white circle: nonpolar organic solvent, and white diamond: polar organic solvent [15].

The chemistry concept of “like dissolving like” is the basic principle underlying the organic solvent-based extraction of microalgal lipids.  Figure 3 illustrates the principle of the 5-step-microalgae lipid extraction mechanism. Typically, the organic solvents penetrate through the cell membrane into the cytoplasm (step 1) and interact with the lipid complex (step 2). During this process, the nonpolar organic solvent interacts with NLs through van der Waals associations, while the polar organic solvent interacts with the polar lipids by generating hydrogen bonds that are strong enough to replace the lipid-protein associations that prevent nonpolar organic solvent from accessing the lipids. Subsequently, an organic solvent-lipids complex is produced (step 3), followed by the organic solvent-lipids complex diffusing across the cell membrane (step 4) and the static organic solvent film (step 5) into the bulk organic solvent driven by a concentration gradient.

The commonly used organic solvent extraction procedures are summarized in Table 1. The nonpolar organic solvents, such as hexane, benzene, toluene, diethyl ether, ethyl acetate, and chloroform, are usually combined with the polar organic solvents to maximize the extraction efficiency of NLs. As such, when lipid extraction is achieved with the use of a nonpolar/polar organic solvent mixture, the polar organic solvent is intended to disrupt the neutral-polar lipid complexes while the nonpolar organic solvent aims to solubilize the intracellular NLs [65]. Moreover, the lipid yields vary with the type of used organic solvents and the ratios of polar solvents to nonpolar solvents. Therefore, the final lipid extraction efficiencies using different organic solvents extraction methods cannot be impartially compared. In addition, the different experiment steps, equipment, and experimental conditions involved in the extraction process also contribute to various extraction results.

The organic solvent-based extraction methods usually require a relatively large quantity of biomass and have few environmental impacts. In addition, organic solvents are not highly selective towards the desired neutral (mono-, di-, and triacylglycerols) lipids and free fatty acid components; some of them are not easily removable, posing difficulties to the subsequent process. An ideal solvent for the lipid extraction should be free of toxicity, easy to remove, and more selective towards target products. These characteristics have been found in CO_2_-based solvents, ionic liquids, and switchable solvents, which will be introduced hereinafter.

#### 4.1.2. CO_*2*_-Based Solvent Extraction

The supercritical (scCO_2_) and liquid (lCO_2_) CO_2_ are able to solubilize many organic molecules and can be easily recycled at the end of the process while leaving no residual solvents, making them promising alternatives to traditional organic solvents.

*(1) scCO_*2*_ Extraction. *The supercritical fluid extraction (SFE) is a promising green technology that can potentially displace the use of traditional lipid extraction procedure, due to its high selectivity, short extraction time, and their absent use of toxic organic solvents [66]. As can be seen from Table 2, scCO_2_ has been regarded with interest in the field of SFEs, because it offers advantages of negligible environmental impact, high diffusivity, no toxicity, no oxidation or thermal degradation of extracts, and easy separation of desired bioproducts [67]. Moreover, scCO_2_ has high selectivity towards microalgal NLs (mono-, di-, and triacylglycerols) and has been used in the lipid extraction of microalgae such as* Cylindrotheca closterium, Arthrospira maxima, Nannochloropsis oculate, Chlorella vulgaris, *and* Spirulina platensis* [68–71]. For example, Halim et al. [72] employed scCO_2_ into a wet* Chlorococum* sp. paste to obtain a yield of 7.1 wt% at a temperature of 333 K and a pressure of 30 MPa over an 80 min extraction time. Moreover, coupling the nonpolar scCO_2_ with the polar cosolvents (i.e., methanol, ethanol, and toluene) could enhance the affinity towards NLs that form complexes with polar lipids, resulting in a greater biofuel production [73]. The general procedure of scCO_2_ extraction is described as follows: CO_2_ is first condensed to lCO_2_ and then to the scCO_2_. Subsequently, the fluid is pumped into the extraction vessel under the desired and controlled conditions of pressure and temperature. After the extraction, the extracted lipids are precipitated and collected into a glass trap, cooled in an ice bath with the amount assessed by gravimetry. It should be noted that the effects of operating conditions (i.e., extraction vessel size and type, pressure, and extraction time) involved in the scCO_2_ extraction process noted above on the lipid yield and selectivity should be investigated on a case-by-case basis. Moreover, the high temperature (i.e., 100°C) and pressure (i.e., 41 MPa) requirement are the main concerns that have limited this approach from industrial-scale application [74].

*(2) lCO_*2*_ Extraction. *Comparatively, lCO_2_ shares many of the same benefits as scCO_2_ while it requires lower temperature and pressure than scCO_2_ extraction (Table 2) and therefore has emerged as a possible substitute. Paudel et al. [75] recovered about 26 wt% of the extractable lipids using lCO_2_ directly from the wet biomass of* Chlorella vulgaris* under different pressures (6.8–17 MPa) and a constant temperature of 25°C. An extraction example using lCO_2_ is described as follows. Firstly, lCO_2_ is pressurized to a certain pressure (i.e., 6.8 MPa) using the high pressure pump and during this process, a coolant (i.e., 75% ethyleneglycol in distilled water) must be used to keep the pump at −5°C to prevent it from being heated. Secondly, the pressurized lCO_2_ is delivered through the tube coil to tube vessel, aiming to make CO_2_ coming from the cold pump warm up to the temperature of the water bath. Thirdly, the vessel containing dry algae is heated in water bath at 25°C for 2 h, during which the required pressure in the system is maintained by backpressure regulator (BPR). After the extraction, the remaining CO_2_ is vented into the flask, and the remaining extract exiting the BPR is captured, separated, and dried. The extract is then preserved in the ice-cold isopropanol.

**Figure 4:**
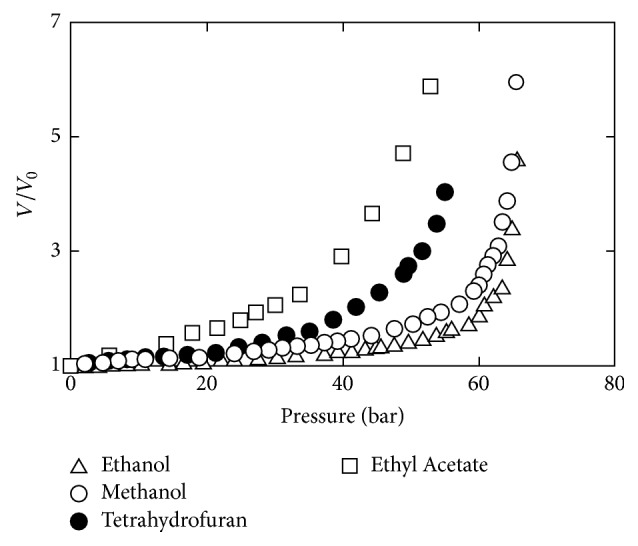
Isothermal volumetric expansion of benign solvents by CO_2_ at 40°C [77].

*(3) Gas Expanded Liquids Extraction. *Gas expanded liquids (GXLs) are liquids expanded in volume by the application of modest pressures with a compressible gas, among which CO_2_ is one of the most commonly used gases [76]. The GXLs are made up of a mixture of compressed gases and conventional solvents. Jessop and Subramaniam [77] reported that GXL solvents have the combined beneficial properties of a compressed gas and organic solvent, so the properties of solvent can be adjusted through variations in the pressure. As can be seen from Figure 4, the gaseous CO_2_ has a considerable solubility in many benign organic solvents at adequate pressures (<8 MPa) such as ethanol, methanol that show a 2- to 3-fold volumetric expansion at relatively mild pressures and moderate temperatures [77]. As such, various principles and applications of CO_2_-expanded liquids (CXLs) including lipid extraction have been proposed [78, 79]. Due to the fact that CXLs can be operated at mild temperatures and pressures, a reduction in process costs and energy consumption could be realized. The mass transfer rates can also be improved via CXLs by reducing interfacial tension and viscosity as well as increasing diffusivity [80]. Wang et al. [78] used the CO_2_-expanded ethanol to successfully extract lipids from* Schizochytrium* sp. with a 35.7 wt% lipid content of dry biomass. The CO_2_-expanded methanol increased up to 82% of the selectivity of methanol towards the extraction of biodiesel-desirable NLs and free fatty acids [75].

#### 4.1.3. ILs Extraction

ILs are organic salts with the melting point below 100°C, and they typically consist of large asymmetric organic cations coupled with smaller anions [81]. Advantages such as thermal stability, synthetic flexibility, nonvolatility, nonflammability, recyclability, and unique solvent properties have made ILs promising replacements of traditional organic solvents in lipid extraction as they can dissolve highly recalcitrant biopolymers [82]. For instance, ILs are capable of disrupting cell structure in wet microalgae biomass under mild conditions. This allows either autopartitioning of the lipids or presumably improving access of cosolvents to the intracellular lipids, thus facilitating the extraction of lipids from microalgae and making it faster than organic solvent extraction processes. Most solvent-based extraction processes, however, are incompatible with wet biomass, which add significant costs to the overall process since dewatering and drying processes are thought to be responsible for up to 70% of the biofuel production cost [83].

A typical lipid extraction procedure with the aid of ILs is described as follows [84]. Firstly, mix microalgae paste with 1 : 10 mass ratio of dry equivalent microalgae to [C_2_mim] [EtSO_4_] and incubate the mixture. Secondly, add water to the mixture to improve separation after the addition of hexane and remove the top layer to a new container. The procedures noted above are repeated three times to achieve a high extraction efficiency. After the extraction, wash the extracts with NaCl and transfer the target part to a preweighed vessel. The mass of extractable lipids is measured after evaporating the solvent. For ILs recycling, add methanol/water to the mixture to precipitate the residual solids and pool hexane with the previous extraction. Subsequently, filter the solvent and wash with methanol/methanol to collect ILs, and then ILs are recovered by evaporation.

#### 4.1.4. Switchable Solvents Extraction

**Figure 5:**
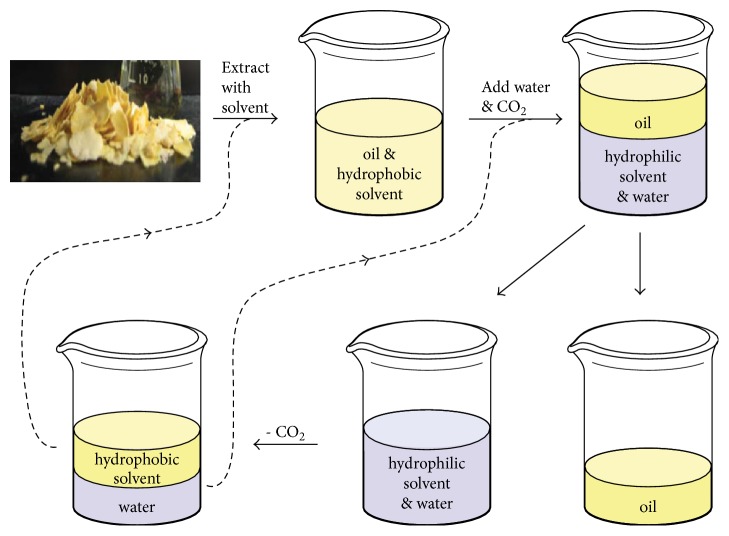
The process of SHSs used for soybean oil extraction from soybean flakes without a distillation step. The dashed lines indicate the recycling of the solvent and the aqueous phase [88].

Switchable solvents, known as “reversible” or “smart” solvents, can reversibly change their properties upon addition or removal of a “trigger.” The switchable solvents (subclass of ILs) are divided into two categories, switchable polarity solvents (SPSs) and switchable hydrophilicity solvents (SHSs) [85, 86]. Specifically, the polarity of SPSs exhibits variation with the solution CO_2_ concentration. The polarity of the solvents can be reversed by removing the CO_2_ from the system by heating or sparging the solution with nonacidic gases. SPSs are divided into two classes, which are either single-component or two-component species. In the two-component SPSs, a base with an alcohol or with an amine is usually included while single-component SPSs require a primary or secondary amine [85]. Unlike SPSs, SHSs can change from a hydrophobic solvent into a hydrophilic one, and their potential applications have extended to the extraction of microalgal lipids [87]. In the SHSs system, the hydrophobic form creates a biphasic mixture with water, and the hydrophilic form is the corresponding bicarbonate salt; thus SHSs could also be reversibly converted between the above two forms by the addition or removal of CO_2_ [88, 89]. A proposed lipid extraction procedure using SHSs is illustrated in Figure 5. Briefly, SHSs are employed to dissolve or extract lipids in their hydrophobic state; the carbonated water is introduced to revert SHSs's hydrophilic form and form a two-phase of the lipid and SHSs/water; finally, the SHSs/water mixture would be separated into two components and then be reused by flushing air through [88].

### 4.2. Nile Red Lipid Visualization Method

Compared with the mostly used gravimetric methods noted above, Nile red lipid visualization method is more convenient as the number of samples and preparation time are greatly reduced. The Nile red (9-diethylamino-5H-benzo[*α*]phenoxazine-5-one) is a lipid-soluble probe that fluoresces at the defined wavelengths depending upon the polarity of the surrounding medium. However, due to the composition and structure of the thick and rigid cell walls in some microalgae species, Nile red is prevented from penetrating the cell wall and cytoplasmic membrane, and therefore, lipids cannot provide the desired fluorescence. Thus, the dimethyl sulfoxide (DMSO) is introduced to microalgal samples as the stain carrier at an elevated temperature [63]. When microalgal lipids are measured by Nile red visualization method, a standard curve preparation is included in most cases.  Table 3 summarizes some specific Nile red lipid procedures for the determination of microalgal lipids. Different solvents are combined with Nile red solution to stain microalgal culture samples, and the samples are diluted if necessary. The lipid content determination is achieved by comparing the resulting fluorescence values to a certain standard curve, in which the wavelength of excitation and emission may be different. Nevertheless, the lipid contents measured by this method are usually interfered by the environmental factors and other components in the cell cytoplasm, and the fluorescence intensity varies between samples. Thus, the optimal spectra and reaction conditions should be determined for each type of sample prior to the fluorescent measurement [90].

### 4.3. SPV Method

The colorimetric SPV method is a rapid alternative for lipid measurement because of its fast response and relative ease in sample handling [91]. The SPV reacts with lipids to produce a distinct pink color, and the intensity is quantified using spectrophotometric methods; therefore, it is employed for direct quantitative measurement of lipids within liquid microalgal cultures [40]. However, the results of SPV assay can be affected by lots of factors such as the degree of oil saturation, incubation time, heating, and cooling; thus the SPV assay may give misleading results [27].

The general procedure of SPV method includes the sample addition, solvent evaporation, sulfuric acid addition, samples incubation, color developing by adding phosphovanillin reagent, absorbance reading, and measurement of the lipid content based on the standard curve [62]. Phosphovanillin reagent is prepared by dissolving vanillin in absolute ethanol and DI water, followed by the addition of concentrated H_3_PO_4_. To prepare standard lipid stocks, canola oil is firstly added to chloroform, and then different amount of standard lipid stocks is added to the tubes. After that, these tubes are treated to evaporate the solvent followed by the addition of water. Subsequently, these samples are prepared by following SPV reaction methods: (1) suspend tested samples in water and place in a glass tube; (2) add concentrated sulfuric acid followed by heat treatment and ice bath; (3) add freshly prepared phosphovanillin reagent and incubate in incubator shaker; (4) read absorbance at 530 nm and determine the lipid content by comparing to the standard curve.

### 4.4. TLC Method

TLC is also a promising alternative to conventional lipids measurement approaches as it requires minimal equipment which is available in most laboratories, and it can also provide additional information about lipid classes, which is important for biofuel production [92]. Among different solvent systems, the multi-one-dimensional TLC (MOD-TLC) separates the lipid classes rapidly and reproducibly. The MOD-TLC method can achieve the quantification for the majority of microalgal lipids through modifications in solvent mixtures and lengths of separation times, and the mass of each resolved lipid band is determined by comparing band intensities of unknown samples (visualized by the lipophilic dye primulin followed by an automated laser-fluorescence detector scanning) to dilution curves of authentic standards. Compared to two-dimensional thin-layer chromatography, MOD-TLC directly analyzes multiple samples on a single TLC plate, while still providing good resolution for the quantification of most major classes of lipid species [32]. Some TLC running procedures are introduced in detail (Table 4). Usually, TLC plates must be activated before TLC running, and NLs are separated by certain solvents such as a mixture of chloroform : methanol : acetic acid : water (85 : 12.5 : 12.5 : 3, v/v/v/v). The determination of microalgal lipid content is finally achieved by comparing the resulting fluorescence values with a standard curve.

## 5. Quantification for Microalgal Fatty Acids

The theoretical biofuel potentials of microalgal biomass are ultimately determined by the acyl chains of the lipids, and therefore the lipid contents are quantified as the sum of their fatty acid constituents. The fatty acids constituents vary with their structural features such as chain length, degree of saturation, branching of carbon chain, positional isomers, configuration of double bonds, or other chemical groups (i.e., hydroxy, epoxy, cyclo, and keto) [93]. It was reported that C16 and C18 are the most abundant microalgal fatty acids including palmitic acid (hexadecanoic, C16:0), stearic acid (octadecanoic, C18:0), oleic acid (octadecenoic, C18:1), linoleic acid (octadecadienoic, C18:2), and linolenic acid (octadecatrienoic, C18:3). The other fatty acids such as C14, C20, and C26–C32 are relatively in low concentrations [94].

Table 5 summarizes and compares different methods of the measurement and quantification of microalgal fatty acids. These methods consisted of two steps: (1) the preparation of FAMEs; (2) quantification and composition analysis of FAMEs by gas chromatography (GC). In the first step via the transesterification or in situ transesterification process, the triglycerides contained in algal lipids are reacted with methanol to produce FAMEs and glycerol. Catalysts (acid catalyst, base catalyst, or the mixture) and heat (water or oil bath) are usually required during the transesterification process to speed up the reaction. The common catalysts for this transesterification include NaOH [95], HCl [35], H_2_SO_4_ [37], acetyl chloride/methanol (1 : 10, v/v) [42], and a mixture of methanol, H_2_SO_4_, and chloroform (1.7 : 0.3 : 2.0, v/v/v) [47]. In the second step, the separation of FAMEs from the mixture and their quantification are performed using GC. The procedure noted above is based on the amount of fatty acids after the lipid extraction in algal biomass. Comparatively, the in situ transesterification is a relatively simpler process and achieves the transesterification to get FAMEs directly from the whole biomass with no requirement of lipid extraction. Therefore, it is able to obtain all fatty acids in the biomass and accurately represent the reflection of biofuels potential [49, 96]. In addition, various procedures for transesterification are followed in terms of microalgal species, lipid contents, and targeted FAMEs fraction. The specific transesterification procedures performed in literature are listed in Table 5. In brief, the lipid extracts or the algal biomass are mixed with the catalysts and methanol and are reacted at the conditions of high temperature. The produced FAMEs are then recovered in solvents like hexane for further purification and quantification. Subsequently, the purified FAMEs are separated and analyzed by GC equipped with the flame ionization detector (FID) and specific columns running at various temperatures. The identification and quantification standards are required such as the commercial 37-component standards, pentadecanoic acid, and heptadecanoic acid [40, 41, 47, 48, 97]. Specific FAMEs are quantified by comparing their peak areas with those of the standards. It should be noted that there is still not a routine method for the quantification of fatty acids specific to algal biomass issued by Association of Analytical Communities (AOAC) International. All the methods noted above vary significantly including the procedures, types of used chemicals and their doses, and analytical apparatus. This might result in a lack of comparability between FAMEs concentrations obtained from different methods.

## 6. Conclusion

Microalgae have proven to be one of the most promising feedstocks for the production of third-generation biofuels that are both economically feasible and environmentally sustainable. Rapid, accurate, sustainable, and cost-effective methods for the lipid extraction and quantification are essential for the rational application of microalgae-based biofuel production. Gravimetric method is most widely used but requires quite a few amount of samples; Nile red lipid visualization method is rapid as the number of samples and preparation time are greatly reduced while a correlation between fluorescence and lipid levels must be previously established as the cell staining varies among different microalgae species; the results of SPV assay can be affected by lots of factors such as the degree of oil saturation, incubation time, heating, and cooling; thus the SPV assay may give misleading results; in addition to the quantitative measurement of microalgal lipids, TLC can also provide additional information about lipid classes which is important for biofuel production. Various lipid quantification methods could be considered on a case-by-case basis, but more effective and greener techniques (e.g., CO_2_-based methods) for microalgal cell disruption and extraction are still required to maximize lipid yields while avoiding the issues of toxicity, flammability, and time consumption for extraction. In addition, the identification and quantification of fatty acids in extracted lipids are also important to evaluate the quality of microalgae-derived biofuel, during which transesterification and in situ transesterification are involved in the preparation of FAMEs for the further the quantification and composition analysis of FAMEs by GC. It is worth mentioning that* in situ* transesterification is relatively simpler and more convenient as it does not require lipid extraction, thus achieving the transesterification to get FAMEs directly from the whole biomass.

## Figures and Tables

**Table 1:** Determination of microalgal lipid content using conventional organic solvents.

Lipid extraction		Determination of lipid content		Ref.
Reagent(s)	Extraction process	Treatment for final determination	Expression of lipid content	
Chloroform : isopropanol (1 : 1, v/v) andhexane.	Add solvent mixture to frozen pellets; centrifuge and transfer supernatants; reextract pellets with hexane and centrifuge to collect supernatants.	Dry the combined supernatant in a speed vacuum and record the weight.	A percentage of total fresh weight (% w/w)	[21]

Ethyl ether.	Ground dry samples to powder and use the Soxhlet extractor.	Distill the solvent, dry the residue, and record the weight.	A percentage of dry cell weight (% w/w)	[22]

Methanol : chloroform : 1% NaCl (2 : 2 : 1, v/v/v).	Extract the biomass with the solvent mixture.	Evaporate the chloroform layer; dry the sample and record the weight.	The weight ratio of extracted lipid to lyophilized pellets (% w/w)	[23]

Deionized (DI) water : methanol : chloroform (1 : 2 : 1, v/v/v); chloroform.	Add solvent mixture to harvested biomass and react for 24 h; mix and then add solvent to achieve a final DI water : methanol : chloroform ratio of 0.9 : 1 : 1; centrifuge, remove, and filter the lipid-chloroform layer; repeat the above steps for second extraction.	Evaporate the chloroform layer; cool the tube and record the weight.	A percentage of total biomass weight (%, w/w)	[24]

Chloroform : methanol (2 : 1, v/v).	Mix dry algal powder with the solvent mixture; put under water bath with the aid of ultrasound; centrifuge and repeat the above steps three times.	Evaporate the organic solvent and weight.	A percentage of total biomass weight (%, w/w)	[25]

Chloroform : methanol (2 : 1, v/v).	Extract lipid from dry biomass with the solvent mixture.	Evaporate the solvent and weight.	Weight difference between the blank flask and the flask containing the extracted oil	[26]

**Table 2:** Comparisons between conventional organic solvents extraction and CO_2_-based solvents extraction approaches.

Items	Organic solvent	scCO_2_	lCO_2_
Heavy metal contamination	Unavoidable	Free of heavy metals	Free of heavy metals
Inorganic salt content	Difficult to avoid	Free of inorganic salts	Free of inorganic salts
Selectivity	Poor selectivity	Highly selective	Highly selective
Extracted compounds	Polar and nonpolar compounds	Nonpolar compounds	Nonpolar compounds
Safety	Flammable and/or toxic	Nontoxic and nonflammable	Nontoxic and nonflammable
Operation condition	Regular temperature and pressure	High temperature and pressure	Lower temperature and pressure than scCO_2_
Recycling	Solvent recovery is expensive	CO_2_ could be recycled and reused	CO_2_ could be recycled and reused
Operation cost	High power consumption (in solvent recovery)	High power consumption	Lower than scCO_2_
Extraction time	Time-consuming	Shorter than solvent extraction	Shorter than solvent extraction

**Table 3:** Nile red assay procedures for the determination of microalgal lipids.

Reagent(s)	Nile red lipid assay procedure	Fluorescence determination	Ref.
Isopropyl alcohol (IPA); Nile red solution; bleach solution; methanol; corn oil (dissolved in 2 : 1 methanol/chloroform).	Suspend lipid extracts in chloroform; dilute extracts with methanol; add diluted samples and corn oil to the microplate to achieve a range; incubate the plate and evaporate the solvents; add IPA and cool the plate; add Nile red solution; add bleach solution to each well; incubate the plate.	Determine fluorescence using a plate reader with excitation set to 530 nm and emission set to 575 nm with a 570 nm cut-off; read the plate; use the first reading after the fluorescence peak for quantitation.	[27]

DMSO; Nile red solution; triolein.	Place microalgal cells in microcentrifuge tubes and put under microwave treatment; mix with DMSO; put under second microwave treatment using the previous conditions; add Nile red solution and incubate the tubes in the dark; pipet the samples into 96 well microplates.	Measure fluorescence using Fluorescence Analyzer with excitation at 535 nm and emission at 580 nm; measure untreated suspension and medium containing Nile red alone as autofluorescence; convert fluorescence to dry weight of lipids.	[28]

DMSO; Nile red solution; virgin olive oil.	Stain diluted microalgal culture samples with Nile red using DMSO as carrier; top up the volume and incubate with agitation.	Read the fluorescence using Microplate Reader with excitation set to 520 nm and emission captured at 570 nm; compare the fluorescence to a virgin olive oil standard curve.	[29, 30]

Pure triolein; chloroform; isopropanol; Nile red stock (in 100% spectral grade acetone).	*Standardized sample fluorescence*: add triolein to chloroform and dilute with isopropanol; prepare working standards by bringing intermediate stocks with DI water. *Nile red assay*: add Nile red to cell suspension or to a lipid standard.	Measure fluorescence using spectrophotofluorometer with excitation set to 475 nm and emission set to 580 nm; express cellular neutral lipid as triolein equivalents.	[31]

**Table 4:** Processes of TLC running for the measurement of microalgal lipid content.

Preparation for TLC analysis	TLC analysis	Ref.
Prepare the solvent by shaking and degassing; add solvents to the TLC tank and equilibrate TLC tank.	Add samples to plates; dry the samples and run the plates in hexane : diethyl ether : acetone (60 : 40 : 5, v/v/v); dry plates and run in chloroform : methanol : ammonium hydroxide : water (60 : 35 : 4 : 1, v/v/v/v) to 18 cm; score plates and break apart with hand pressure; determine the line on a different plate or spray only the outside lane of the plate to be cut; rotate the lower portion by 180° and run in chloroform : methanol : acetic acid : water (85 : 12.5 : 12.5 : 3, v/v/v/v) in the original orientation; dry the plates and spray with a solution of primulin dye; visualize lipid spots and scan the plates by laser-excited fluorescent detection; quantify spots and compare to standard curves.	[32]

Elute silica gel with chloroform; elute lipids with chloroform to yield NLs; dry NLs and re-suspend in chloroform; activate plates in an oven.	Subject the NL fraction to TLC for lipid class separation and identification; use hexane/diethyl ether/acetic acid (70 : 30 : 1, v/v) for lipid separation; co-chromatography with pure standards; stain bands of lipid classes with 2, 7-dichlorofluorescein; visualize under UV light.	[33]

Elute silica gel with chloroform; elute lipids with chloroform to yield NLs; dry NLs and resuspend in chloroform; activate plates in an oven.	Use 80 : 20 : 1 hexane/diethyl ether/acetic acid for lipid separation; spray the plate with a solution of primulin dye; illuminate the plate with UV light.	[27, 32, 33]

**Table 5:** Methods employed for determining fatty acids after lipid extraction, including the transesterification and in situ transesterification. Both methods consist of the preparation of FAMEs and the quantification and composition analysis of FAMEs by GC.

Method	Preparation of FAMEs	GC operation	Standards used	Ref.
Transesterification	Redissolve total lipid extracts and elute neutral lipids and polar lipids with different solvents; after drying under nitrogen gas, derivatize to FAMEs and recover the FAMEs.	GC equipped with FID and Agilent CP-Wax 52 CB column.	(1) Internal standard: tripentadecanoin, C15:0 triacylglycerol, Sigma-Aldrich. (2) Identification and quantification standard: Supelco® 37-component standards.	[34]
	Add HCl and methanol to lipid extracts and heat mixture with hexane and methyl-tert-butyl ether; wash the upper organic phase with sodium hydroxide; aspirate two-thirds of the organic extracts and transfer to a sample vial.	GC equipped with FID and a special performance capillary column (Hewlett Packard model #HP-5 MS).	(1) Internal standard: hexadecane (Sigma-Aldrich #H6703) standard. (2) Calibration standard: olive oil.	[35, 36]
	Add H_2_SO_4_ to lipid extracts and heat; cool the sample to room temperature and mix with DI water to separate lipid extracts; move the lower part liquid into a vial.	GC equipped with FID and a Supelco NucolTM column (355 33-03A, film thickness) using Helium as the carrier gas (flow 20 mL·min^−1^).	(1) Internal standard: pentadecanoic acid (C15:0). (2) Identification standard: authentic standards (Sigma-Aldrich, MO, USA).	[37, 38]
	Add methanolic HCl to lipid extracts and flush headspace with nitrogen and seal tightly and heat; cool vials and add aqueous K_2_CO_3_; centrifuge and remove the upper phase and dry.	GC equipped with FID and a SGE Sol Gel-WaxTM capillary column using helium as the carrier gas.	(1) Identification standard: standard fatty acids (Nu-Chek Prep Inc., Elysian, MN). (2) Quantification standard: heptadecanoic acid (C17:0).	[39]
	Add chloroform containing heptadecanoic acid (C17:0), methanol, and sulfuric acid to each tube and heat; cool down, add DI, centrifuge, and separate the lower chloroform phase and filter for test.	GC equipped with FID and HP19091N-213 HP-INNOWax polyethylene glycol column.	(1) Internal standard: heptadecanoic acid (C17:0). (2) Identification and quantification standard: 37 component FAME standard mix (Supelco, Bellefonte, USA).	[40]
	Add BHT (butulated hydroxy toluene, 1% in methanol) to prevent oxidation prior to methylation.	GC equipped with FID and the capillary column DB-23 (Agilent Technologies) using helium as the carrier gas (1 mL·min^−1^, splitless).	(1) Quantification standard: C19:0 (nonadecanoic Acid 72332-1 G-F/analytical standard, Sigma-Aldrich). (2) Identification standard: Supelco TM 37 Component FAME Mix, Sigma.	[41]
	Add freshly prepared acetyl chloride/methanol (1 : 10), methanol into lipid extracts, and seal vials and heat; cool down and add K_2_CO_3_, hexane; centrifuge the samples and recover the hexane supernatant.	GC equipped with FID and a BPX70 capillary column (120 m × 0.25 mm internal diameter, 0.25 *μ*m film thickness, SGE Analytical Science, Ringwood, VIC, Australia) using helium as carrier gas (1.5 mL·min^−1^).	(1) Internal standard: C23:0. (2) Identification standard: a series of mixed and individual standards from Sigma-Aldrich.	[42, 43]
	Mix H_2_SO_4_, methanol, THF, and lipid extracts; reflux the reaction mixture at 90°C with continuous stirring for 3 h; neutralize the mixture with NaHCO_3_ and extract it with hexane.	GC equipped with FID and HP-INNOWax column (30 m × 320 *μ*m × 0.25 *μ*m film of polyethylene glycol) using helium as carrier gas.	(1) The standard FAMES: C14:0, C16:0, C16:3, C18:0, C18:1, C18:2, C18:3, and C20:0. (2) Supelco TM 37 Component FAME Mix, Sigma-Aldrich.	
	Treat extracted lipids with methanolic sulfuric acid and heat; recover FAMEs in hexane; centrifuge the suspension and aspirate hexane, containing FAMEs, into new glass tube.	GC equipped with DB-5 capillary column (30 mm : 0.25 mm : 1 *μ*m) and FID using helium as carrier gas (1 mL·min^−1^).	NISTIL.S database.	[44–46]

In situ transesterification	Add a mixture of methanol, sulfuric acid, and chloroform into dried cell biomass and heptadecanoic acid (C17:0) as an internal standard and heat; cool down, add DI water, mix, and settle; transfer the lower phase containing FAMEs to a clean vial and dry with anhydrous Na_2_SO_4_.	GC equipped with FID and SGE Sol Gel-WaxTM capillary column (30 m × 0.25 mm × 0.25 *μ*m) using helium as the carrier gas.	(1) Identification standard: standard fatty acids (Sigma, MO). (2) Quantification standard: internal standard (C17:0).	[47, 48]
	There are many different ways to add a catalyst, such as methanolic hydrogen chloride, NaOMe and BF_3_, NaOMe, and tetramethyl guanidine and methanol; tridecanoic acid methyl ester (C13-FAME) as an internal standard and heat.	GC equipped with FID (Agilent 6890N, HP 5-MS column (Agilent, USA), 30 m 0.25 mm ID and 0.25 *μ*m FT) using helium as the carrier gas (1.5 mL·min^−1^).	(1) C8–C24, SIGMA cat #18918 C13–C21, SIGMA cat #1896.	[49]

*Note*. (1) All samples prepared for FAMEs contain the corresponding internal standards that have been listed in the table above. (2) FID: flame ionization detector.
